# Supplementary material for: Chromothripsis during telomere crisis is independent of NHEJ, and consistent with a replicative origin
Source: Genome Res. 2019 May;29(5):737–49. doi: 10.1101/gr.240705.118 (PMC6499312; doi:10.1101/gr.240705.118)
Supplement: Supplemental Material [file supp_gr.240705.118_Supplemental_file_1.zip › contigs/annotated_contigs/DB96/contig.2.DB96_length_296_mean_cov_4.2027027027.docx]

**DB96_length_296_mean_cov_4.2027027027**

```
TTTGCTATTTTTCCCTGGTATTTTGCTGGGTGTTATAAGGAAGTGTGAAACAAGAAATGTGTTTTGCCTGTACCAAACATTTGGTTGTA
 >chr5:132245607-132245844 - E=1e-128
GAAATCAATAATACAAAAGAAAGCAATAGGAATTATTTATTAGTGTTTAATTGGATGTGTAGTTGTGTGCATCACACTTAGACGTAAGT

TGTCTTCTCTTTGTATTCTGTTCTTTACCTAGAACCTCTTTACCTAGGTGCCTCAC|AAA|AAAAAAAATTACAAAAACTAACTGGTTT
                                                         >chrUn_gl000220:143704-143766
CATAACCTGGACTCAAAGTTAATAAATAGAT- E=6e-26
```
